# Exploring the mechanical and biological interplay in the periodontal ligament

**DOI:** 10.1038/s41368-025-00354-y

**Published:** 2025-04-02

**Authors:** Xinyu Wen, Fang Pei, Ying Jin, Zhihe Zhao

**Affiliations:** State Key Laboratory of Oral Diseases & National Clinical Research Center for Oral Diseases & Dept. of Orthodontics, West China Hospital of Stomatology, Sichuan University, Chengdu, China

**Keywords:** Cell biology, Structural biology

## Abstract

The periodontal ligament (PDL) plays a crucial role in transmitting and dispersing occlusal force, acting as mechanoreceptor for muscle activity during chewing, as well as mediating orthodontic tooth movement. It transforms mechanical stimuli into biological signals, influencing alveolar bone remodeling. Recent research has delved deeper into the biological and mechanical aspects of PDL, emphasizing the importance of understanding its structure and mechanical properties comprehensively. This review focuses on the latest findings concerning both macro- and micro- structural aspects of the PDL, highlighting its mechanical characteristics and factors that influence them. Moreover, it explores the mechanotransduction mechanisms of PDL cells under mechanical forces. Structure-mechanics-mechanotransduction interplay in PDL has been integrated ultimately. By providing an up-to-date overview of our understanding on PDL at various scales, this study lays the foundation for further exploration into PDL-related biomechanics and mechanobiology.

## Introduction

Periodontal ligament (PDL) is the connective tissue that connects the cementum to the alveolar bone.^1,2^ It supports the teeth as well as transmits and disperses the biting force.^3^ In addition, the PDL is closely related to maintaining the correct position of the mandible and tissue repair.^1,4^ In orthodontic treatment, the PDL transmits orthodontic forces and influences bone remodeling.^5,6^ Changes in periodontal blood flow trigger osteoclast differentiation on the compressive side for bone resorption and osteoblast differentiation on the tensile side for bone deposition. Therefore, PDL plays an important role in physiological processes such as mastication and orthodontic treatment. The PDL is composed of cells, fibers, matrix, blood vessels, and other components, which affect the biomechanical properties of PDL.^7–9^ Among them, collagen fiber is the main component, especially type I collagen fiber.^10,11^ The intricate structure and composition of the PDL shape its biomechanical behavior, closely intertwined with the surrounding biomechanical environment.^12^

Heterogeneity exists within the PDL,^13^ not only in the dense collar area and furcation area of the PDL but also within the collar area itself.^14^ This characteristic is related to the unique structural composition of PDL, especially the distribution of collagen fibers. Scholars have developed various constitutive models to describe the nonlinear mechanical properties of the PDL, which include elastic and viscous components.^15^ In addition, viscoelastic and hyper-viscoelastic models have been proposed to capture both the instantaneous elastic behavior and the time-dependent viscoelastic behavior of the PDL.^16,17^ Additionally, a hyper-elastic constitutive model based on the distribution of collagen fibers has been established.^18^ The existence of pores also affects the PDL’s response, prompting researchers to incorporate a porous fibrous structure into hyper-elastic models.^19^ Appropriate and accurate constitutive models are essential for the application of mathematical simulation methods like finite element analysis to evaluate PDL properties.^20^

External mechanical stimulation induces biological effects in periodontal ligament cells (PDLCs) such as aseptic inflammation and tooth movement when stimulated.^21,22^ Many molecule mechanisms are involved in biomechanical signal transduction, with yes-associated protein (YAP) identified as a significant mechanical sensing regulatory factors by scholars.^23^ Micro-RNAs (miRNAs), including miR-34a and miR-146a, also participate in mechanotransduction by inhibiting osteogenic differentiation of periodontal ligament stem cells (PDLSCs) through targeting CELF3.^24^ Understanding these mechanisms is crucial for minimizing tissue damage during orthodontic treatment.^25^

This study integrates the structural characteristics, mechanical properties, and biomechanical transduction mechanisms of PDL, building on existing research to provide novel insights into the mechanical and biological interplay of PDL.^1,12,26–28^ It references the recent research from the past five years to update the knowledge in this field. By elucidating the biological and mechanical characteristics of PDL separately, and then integrating them, we enhance understanding of the biomechanical properties of PDL. In this review, the macrostructure, cell and matrix composition, and nano-microstructure of PDL were summarized. We then discussed the mechanical properties of the PDL, including elasticity, stiffness, viscoelasticity, poroelasticity, and compressibility. Especially, the factors affecting the mechanical properties of PDL from both internal and external dimensions were illustrated. And the constitutive models of PDL in recent years were reviewed. Finally, the mechanotransduction mechanisms of PDL were concluded. This review aims to provide a better understanding of the biological and mechanical characteristics of the PDL. It provides a complete description of current macroscopic and microscopic cognition of PDL, laying the foundation for further study of PDL related biomechanics and mechanobiology.

## Macrostructure and composition

The periodontal ligament (PDL) is a soft and special connective tissue, located between the cementum and alveolar bone at the tooth root.^29^ It typically ranges in width between 0.15 ${\mathrm {mm}}$ and 0.38 ${\mathrm {mm}}$, with its narrowest point usually found in the middle third of the tooth root.^1^ Mechanical forces, especially in the buccal cervical region and collar area, can lead to a reduction in PDL thickness.^30^

**Fig. 1:**
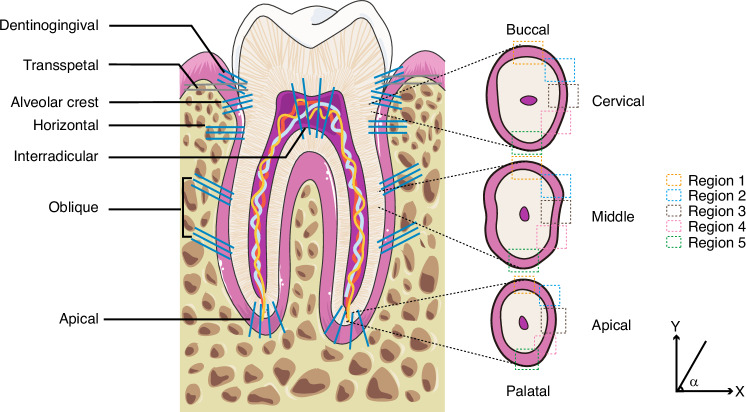
Schematic illustration of PDL principal fiber distribution. The principal fibers can be divided into dentinogingival, transseptal, alveolar crest, horizontal, oblique, apical, and interradicular group according to the direction of fibers

Collagen fibers are essential components of the PDL, with type I collagen constituting a significant proportion of their composition.^31,32^ These collagen fibers form fibrils, which further cross-arranges into principal fiber bundles within the PDL.^33^ Scanning electron microscope (SEM) and transmission electron microscope (TEM) results show that the PDL collagen fibers and fibrils in humans measure approximately 3.1 ${\upmu}{\rm{m}}$ and 90 ${\mathrm{nm}}$, respectively.^23^ In contrast, studies in rats report these dimensions as 1.7 ${\upmu}{\rm{m}}$ and 70 ${\rm{nm}}$.^23^ Within the PDL space, the fibers predominantly run radially but gradually transition to a circumferential direction near the adjacent cementum and alveolar bone.^34^ Circumferential fibers anchor the PDL to the cementum and alveolar bone via Sharpey’s fibers, which have a diameter of 1–2 ${\upmu}{\rm{m}}$, thus ensuring firm tooth stability within the alveolar socket.^35^ In rat models, parameters such as diameter, density, length, and angle of Sharpey’s fibers vary among different tooth positions, specific tooth regions, and different fiber groups.^36^ Notably, the median diameter and length are greater in bone compared to cementum, while the median density is higher on the cementum side.^36^ Based on their position and orientation along the long tooth axis of the tooth, PDL fibers can be categorized into three primary groups: dentinogingival, transseptal, and alveolodental groups.^37^ Dentinogingical fibers run obliquely from the cementum to the gingiva. The transseptal group consists of PDL fibers that traverse the alveolar crest to connect adjacent teeth. The alveolodental group can be further subdivided into the alveolar crest group, horizontal group, oblique group, apical group, and interradicular group (Fig. 1).^26^ The alveolar crest group extends from the alveolar crest to the cervical region of the tooth, while the horizontal group runs horizontally from the alveolar bone to the tooth root. The oblique group is inclined at approximately 45° towards the root, whereas the apical group radiates from the apical cementum to the alveolar bone around the apical region. The interradicular group, found in the furcation region of multirooted teeth, extends from the root furcation to the alveolar bone. Also, it is noted that the embedding angles of Sharpey’s fibers differ among these groups, with the oblique group exhibiting the smallest embedding angles.^36^ Moreover, the orientation of collagen bundles in transverse sections varies among the different groups. The PDL region can be divided into five parts in a transverse view from the buccal to the palatal side (Fig. 1). Regions 1 and 3 exhibit similarities among the cervical, middle, and apical groups, while regions 2, 4, and 5 show variations among these groups. For example, in region 4 of the cervical section, collagen bundles are oriented between –70° and –30°, whereas they range from –50° to –30° in the middle and apical sections. In region 5, the collagen bundles are oriented from 70° to 90° in the cervical section, and from –70° to –50° in the other two sections.^38^

Periodontal ligament is composed of cells and extracellular matrix (ECM).^39,40^ Cell types in the PDL include fibroblasts, osteoblasts, osteoclasts, cementoblasts, odontoclasts, epithelial cells, mesenchymal cells, and immune cells like macrophages.^41,42^ Periodontal ligament stem cells (PDLSCs) are crucial in the PDL,^43–45^ showcasing self-renewal and the ability to differentiate into various cell lineages such as fibroblasts, osteoblasts, cementoblasts.^46–48^ Although most studies hold the view that PDLSCs belong to mesenchymal stem cells (MSCs), distinctions between PDLSCs and MSCs within the PDL may exist.^49,50^ MSCs are associated with tooth development and PDL reconstruction.^51^ In particular, PRX^+^ cells, as a subtype of MSCs, are involved in angiogenesis during molar development and PDL formation in mice.^52^ Macrophages are also distributed in PDL, and their polarization may be related to the immunoregulatory function of PDLSCs.^53^

ECM is a three-dimensional network structure consisting of dynamically changing macromolecules, which supports cells, regulates signal transduction, and forms a microenvironment to maintain cell homeostasis.^54^ Collagen fibers are the principal components within ECM, among which type I collagen is the most abundant.^55,56^ They are arranged in PDL space into well-defined bundles of fibers, namely principal fibers.^57^ Blood vessels and nerves are also significant members of PDL.^58^ Blood vessels benefit nutrient delivery, providing material basis for PDL regeneration and orthodontic tooth movement (OTM).^59,60^ However during OTM, compression of blood vessels in compressive area leads to circulation disturbance and the creation of a local hypoxic environment, thus causing bone resorption.^61^ Nerves are indispensable component of PDL, serving as structure transmitting stress and proprioception. Mechanical stimulation can promote nerve fiber growth.^62,63^

## Microstructure

Recent comprehensive studies on the microstructure of the PDL have revealed that there is heterogeneity of microstructure in PDL. Periodontal ligament fibroblasts (PDLFs) form cellular networks by connecting with neighboring or distant cells, with collagen fiber bundles arranged within the networks.^64^ Advanced 3D visualization techniques like focused ion beam/scanning electron microscope (FIB/SEM) tomography have shown that PDL fiber bundles exhibit a complex multi-branched structure rather than a simple fascicular arrangement.^64^ In different regions of PDL, variations in this structure exist: the horizontal fiber area displays a vertical arrangement of PDLFs and fibers forming a dense multi-branched network, while the oblique and apical groups show fibers aligned in almost parallel and chain-like structures with fewer branches and connections.^64^ The PDL comprises functional regions such as the furcation region and dense collar region, each exhibiting structural heterogeneity.^13^ The collar region resembles typical ligaments with collagen fibers arranged in parallel. Particularly, a unique longitudinal structure rich in type VI collagen and LOX exists in the collar region and increases tissue hardness compared to other areas.^13^

Root morphology and mechanical force influence the heterogeneity of PDL. The distribution of fibers differs between round and kidney-shaped roots with more global fiber distribution in highly curved zones of reniform root.^30^ Under mechanical force, especially dynamic loading, collagen fiber rearrangement and matrix remodeling occur.^65^ Despite macroscopic narrowing of the PDL space under stress, microscopic examination reveals that fibers are stretched rather than compressed, likely due to decreased fluid phase.^30^ Studies using micro-Raman spectroscopy illustrated that orthodontic force duration alters PDL protein structure.^66^ Reduced function and atrophy lead to decreased type I collagen, periosteum protein, and laminin decreased in the PDL, highlighting the effect of mechanical force on its normal structure.^67^ Microvascular imaging shows changes in blood vessel distribution during mechanical stimulation, possibly indicating an initial increase in blood supply followed by adaptation in bone reconstruction.^68^

**Fig. 2:**
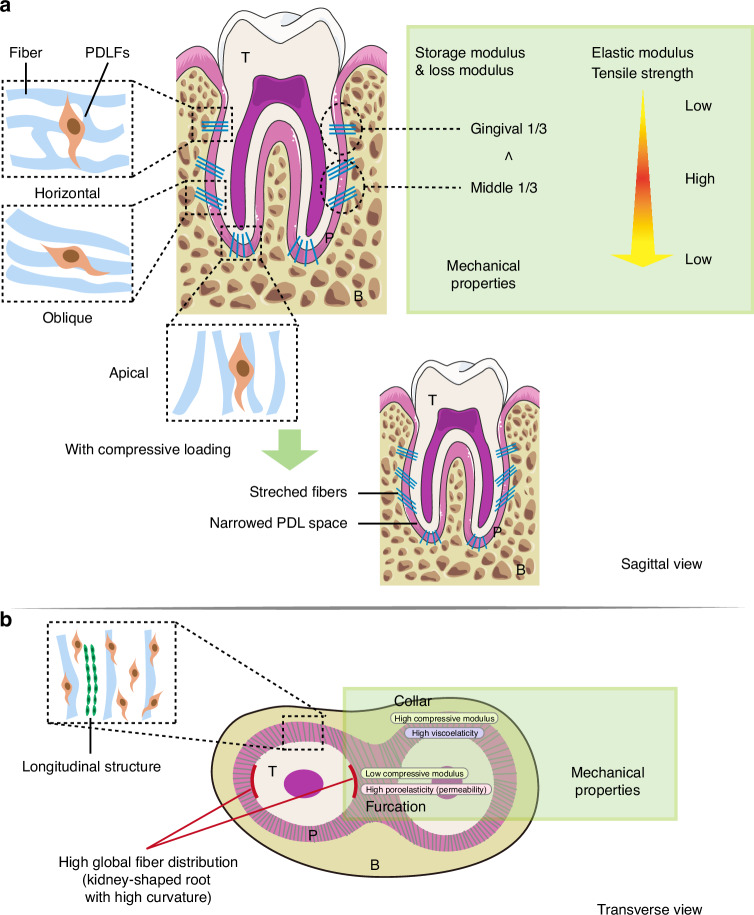
Summary of microstructure and mechanical properties of PDL at sagittal view (**a**) and transverse view (**b**). Green frame in the diagram shows the mechanical properties. T tooth, P periodontal ligament, B alveolar bone

To sum up, there is heterogeneity of microstructure in different PDL zones. Several factors may influence the microstructure of PDL, such as root morphology and mechanical force (Fig. 2).

## Mechanical properties

The mechanical properties of PDL are important for transmitting and dispersing biting force. The interface between the PDL, cementum, and alveolar bone should exhibit a gradual change in properties to avoid stress concentration.^34^ However, in a narrowed PDL space, this gradual change property transition is disrupted, leading to an increased elastic modulus compared to normal PDL spaces. This altered area may become a new load-bearing site, potentially resulting in the fusion of bone and cementum.^69^ Advanced techniques such as nanoindentation have been applied to evaluate the mechanical properties of PDL. Another measurement method utilizing in-fiber Bragg grating (FBG) sensor can assess intact PDL mechanical properties with proven repeatability.^70^ Recent studies have demonstrated that both internal factors, such as the specific regions within individual tooth position, and external factors, including mechanical forces and environmental conditions, significantly influence the mechanical properties of the PDL. Moreover, mathematical methods like constitutive models and finite element analysis have been applied to simulate the mechanical behaviors of PDL. Therefore, this section discusses factors influencing these properties and the use of mathematical simulations. The parameters mentioned for mechanical properties are explained in Table 1.

**Table 1:** Explanation and unit of mechanical property parameters of PDL

Parameter Name	Explanation	Units
Elastic Modulus	The proportional constant of stress and strain in the range of elastic deformation. It shows the ability of a material to resist deformation.	Pa
Compression Modulus	The ratio of compressive stress to compressive strain in a material. It is a measure of a material’s resistance to compression.	Pa
Compressive Strain	The ratio of change in length to the original length of a material when it is subjected to compressive stress.	–
Tensile Strength	The maximum stress that a material can withstand while being stretched or pulled before breaking. It is a measure of the material’s resistance to being pulled apart.	Pa
Dynamic Modulus	The modulus of elasticity of a material under dynamic or oscillatory conditions. It is a complex value that includes both the storage modulus and the loss modulus.	Pa
Storage Modulus	It is the elastic component of the dynamic modulus. It represents the energy stored in a material during deformation and released when the deformation is removed.	Pa
Loss Modulus	It is the viscous component of the dynamic modulus. It represents the energy dissipated as heat in a material during deformation.	Pa
Loss Factor	The ratio of the loss modulus to the storage modulus. It is a measure of the energy dissipated as heat in relation to the energy stored in a material during cyclic loading.	-
Creep Flexibility	The ability of a material to deform over time under sustained stress.	1/Pa
Relaxation Modulus	The ability of a material to reduce stress over time under sustained deformation.	Pa
Poisson’s ratio	It is the negative ratio of transverse strain (lateral deformation) to axial strain (longitudinal deformation) in a material under axial load.	-

### Influence factors of PDL mechanical properties

#### Internal factors: different parts of PDL

The mechanical properties of PDL vary among different regions, with each region matched to specific functions. The collar area exhibits greater stiffness and viscoelasticity under low-frequency loading compared to the furcation area, highlighting its supporting function.^13^ In contrast, the furcation area demonstrates lower stiffness and compression modulus but higher poroelasticity under high-frequency loading.^13^ These findings suggest that the furcation region may serve a dual function. The lower compression modulus indicates increased compressibility, particularly under low-frequency loading, while the higher poroelasticity suggests its ability to resist fluid flow and withstand compression (Fig. 2).^13^ Variations in poroelasticity may be due to tissue permeability and fluid flow, crucial for functions like bearing force and transporting nutrients, while viscoelasticity is associated with the change of collagen and macromolecule.^13,71^ Mechanical properties also differ within the collar area itself. The modulus of buccal PDL is higher than that of lingual side.^14^ Viscoelasticity along the long axis also varies. According to recent research on the PDL of human cadaver maxillary anterior teeth, the middle region exhibited higher tensile strength, while the cervical and apical areas showed lower tensile strength due to a decreased fiber areal fraction.^38^ Additionally, measurements of the storage modulus and loss modulus indicate that the gingival third is less stiff and viscous than the middle third, reflecting a trend of greater stiffness and viscosity in the middle third (Fig. 2).^72^

The variations in mechanical properties across different regions of PDL linked to its internal structure. Studies have shown that compressive strain is greater in the apical area compared to the middle and cervical regions, and that the deformation resistance of human PDL increases with higher fiber content.^7^ Besides of fiber content, the spatial distribution of fibers also plays a significant role in influencing the mechanical properties of the human PDL. When fiber angles are large, tensile resistance decreases.^18^ The heterogeneous nature of PDL structure governs mechanobiological signal transmission and sustains tissue equilibrium.^73^ However, the mechanical properties will alter correspondingly when the tissue homeostasis changes. For example, osmotic swelling within the PDL structure can reduce tension and increase the strength of porcine PDL, resulting in an increase in destructive shear forces.^74^ These results have suggested the relationship between mechanical properties and tissue structure of PDL.

In addition, the mechanical properties of human PDL vary depending on tooth position. For example, under compressive condition, the storage modulus and loss modulus of central incisors are approximately twice as high as those of lateral incisors.^75^ Furthermore, under tensile condition, the dynamic modulus of the central incisor surpasses that of lateral incisors.^72^ Age also influences the mechanical properties of the PDL. Research conducted on rats has shown that shear stress and stiffness may decrease with aging.^76^ However, there is a lack of similar studies on human PDL, likely due to limitations in sample availability. In summary, the mechanical properties of the PDL are influenced by internal factors related to different tooth regions and positions.

#### External factors: mechanical force and environmental parameters

The mechanical properties of the periodontal ligament (PDL) are intricately linked to the type and frequency of mechanical stimulation applied. During compression, the loss factor of the PDL ranges from 0.04 to 0.40, significantly higher than the 0.04 to 0.08 observed during tension, indicating greater viscosity under compressive condition.^77^ At frequencies of 0.05 Hz to 0.5 Hz, both the storage modulus and loss modulus increase with frequency. However, between 0.5 Hz and 5 Hz, the storage modulus continues to rise while the loss modulus remains stable.^75^ Other research indicates that the storage modulus increases from 0.1 Hz to 5 Hz but remains constant from 5 Hz to 10 Hz, with the loss factor showing no frequency dependence.^72^ The storage modulus and the loss modulus reflect the PDL’s elastic and viscous properties, respectively.^75^

Additionally, external factors such as temperature affect PDL mechanics. Recent studies show that the loss factor at 37°C is higher compared to 25°C, implying increased viscosity and reduced elasticity.^78^ Furthermore, occlusal patterns impact the strain distribution and magnitude within the PDL.^79^

It is important to note that some of the studies mentioned above were based on animal experiments involving species such as mice or pigs, which may differ from human PDL. While species may be one of the factors influencing the mechanical properties of the PDL, the trends in variation among regions are similar.^80^ However, species difference should be taken into account in further research.

### Constitutive models and finite element analysis

To gain a comprehensive understanding of the PDL’s mechanical properties, constitutive models and finite element analysis (FEA) are employed to simulate the PDL’s response to various loads.^81,82^

Commonly utilized constitutive models include linear elastic, nonlinear elastic, viscoelastic, and visco-hyperelastic models.^83^ Current research indicates that the mechanical behavior of the PDL is non-linear, anisotropic, and time-dependent.^84^ It is widely accepted that the PDL exhibits viscoelastic characteristics, which has led to the emergence of viscoelastic and visco-hyperelastic models as important areas of research.^85,86^

The PDL demonstrates strain-dependent relaxation, necessitating the use of non-linear viscoelastic models to capture its behavior accurately. Natali et al.^20^ proposed a non-linear viscoelastic model, that describes the mechanical properties of the PDL more precisely. Huang et al.^87^ further advanced this field by developing a visco-hyperelastic model based on nanoindentation experiments, while Liu et al.^88^ constructed and validated a similar visco-hyperelastic model through FEA. Zhou et al.^89^ characterized the PDL as a steady-state rheological non-linear viscoelastic fluid and developed a hyper-viscoelastic model that reflects its complex properties. Although these existing models provide some insight into the biomechanical behaviors of the PDL, many overlook its anisotropic properties, which are crucial for accurate modeling. Research by K. Komatsu et al.^90^ highlights the significance of collagen fiber viscoelasticity in PDL stress–relaxation processes. To account for these factors, Wu et al.^7,18^ created hyper-elastic and viscoelastic models that consider fiber content and distribution along the longitudinal axis of human teeth, noting that the PDL’s resistance to deformation increases with higher fiber content.^7^ Additionally, the porosity and fibrous structure of PDL are pivotal, as certain mechanical properties are influenced by the fluid phase within the PDL. The tension and compression processes behave quite differently: tension is primarily associated with fiber stretch, while compression involves fluid flow into adjacent regions through the PDL’s pores.^91^ It is essential to integrate the solid and liquid phases of the PDL, defining it as a biphasic structure.^92^ To this end, a porous hyper-elastic model has been proposed.^19^ Furthermore, a porous hyper-elastic damage model of the PDL has been constructed by Ortún-Terrazas J et al. to analyze the damage phenomena within the PDL more comprehensively.^93^ These advancements highlight the complexity and necessity of considering multiple factors in modeling the mechanical behavior of the PDL. FEA is extensively used to model tooth forces and movements, particularly under orthodontic loads.^94^ Research has shown that the elastic modulus of the PDL is approximately $9.64\times {10}^{-4}$GPa, as verified by FEA and experimental studies.^95^ However, the accuracy of FEA is contingent on material properties, model parameters, and other factors. Variations in Poisson’s ratio and tension-compression asymmetry can significantly affect simulation outcomes.^96,97^ The integration of response surface methodology with FEA, according to the principle of parameter inversion, is effective in minimizing errors.^98^

To conclude, the PDL is recognized for its viscoelastic mechanical properties. Recent studies have elucidated both internal and external factors influencing these properties. Internal factors include variations across different PDL regions, while external factors encompass loading conditions and environmental temperature. Advances in constitutive modeling and simulation techniques, such as FEA, continue to enhance the understanding of PDL’s mechanical behaviors.

## Biomechanical signal transduction in PDL

As the primary conduit for mechanical force transmission, the PDL conveys various mechanical forces—tensile, shear, and compression—during OTM. These forces are transduced into biological signals, eliciting specific cellular responses with PDL cells playing a crucial role.^99,100^ Mechanical force induces dynamic changes in the proliferative spectrum of PDL cells, leading to tissue remodeling.^101^ For example, fluid shear stress promoted PDLC proliferation in a time-dependent manner through the p38-AMOT-YAP pathway.^102^ In addition, mechanical force regulates osteogenic and osteoclastic differentiation in PDL, leading to corresponding bone formation and bone resorption, realizing bone remodeling and tooth movement. This response differs between adolescents and adults.^103^ Mechanical stimulation also triggers extracellular matrix remodeling in the PDL, mediated by matrix metalloproteinases (MMPs) and their inhibitors.^40^ Clinical studies have further substantiated that mechanical stimulation can alter the gene expression of PDL cells, such as CPNE3, OPHN1, and PPM1F.^104^ Thus, mechanotransduction is pivotal in the interplay between the biology and mechanics of the PDL.^105^ In the context of sensing mechanical stimuli, the biological properties, mechanical properties, and biomechanical signal transduction are three key components of this process. The biological structure of the PDL determines its fundamental mechanical properties, and changes in these mechanical properties influence the function and remodeling of the PDL through biomechanical signal transduction.^106^ This interplay among structure, mechanics, and mechanotransduction forms a complex network of PDL responses to mechanical stimuli which is essential for the effectiveness of orthodontic treatment. To facilitate a deeper understanding of this interplay, we discuss the detailed mechanisms of mechanotransduction here (Table 2).

**Table 2:** Mechanisms of mechanotransduction when compressive, tensile, and other mechanical force are loaded on periodontal ligament

Cell Type	Mechanical Force	Effect	Mechanism	Animal model	Reference
PDLF	Compressive force	Morphology changes, higher cell death rate, reduced cell proliferation	–	–	^107^
PDLSC	Compressive force	Inhibited cell proliferation	Downregulation of MIR31HG via DNA methylation	–	^108^
PDLC	Intermittent compressive force	Osteogenic differentiation	YAP	–	^109^
PDLC	Static compressive force	Osteoclast differentiation	YAP-GDF15 regulation mechanism	–	^110^
PDLF	Compressive force	Reduced inflammation and osteoclastogenesis	HSP27 phosphorylation	–	^111^
PDLF	Continuous compressive loading	Osteoclastogenesis	PIEZO1	Male C57BL/6 N mice	^113^
PDLF	Static compressive loading	PDLF apoptosis	PIEZO1	Male Wistar rats	^114^
PDLSC	Compressive force	Induced proliferation, reduced osteogenesis, improved macrophage migration, improved osteoclsatogenesis	TRPV4 activation	Male Sprague-Dawley rats	^116^
PDLSC	Continuous compressive loading	M1 macrophage polarization	H2S production, STAT1 signaling pathway	Male C57BL/6 mice	^117^
PDLSC	Compressive force	M1 macrophage polarization	PDLSC autophagy, AKT signaling pathway	Male Sprague-Dawley rats	^118^
PDLC	Static compressive force	PDLC autophagy	Upregulated ILK expression in a PI3K dependent manner	–	^119^
PDLSC	Static compressive force	PDLSC autophagy	Upregulated lncRNA FER1L4, AKT/FOXO3 signaling pathway	Male BALB/c mice	^120^
PDLF	Static compressive force	M1 macrophage polarization	YAP-TEAD axis	–	^121^
PDLSC	Compressive force	Osteoclast differentiation	Exosome ANXA3 protein	Male C57BL/6 mice	^123^
PDLC	Continuous compressive loading	Increased inflammation	Th17 cell polarization	Male C57BL/6 J mice	^124^
PDLSC	Mechanical tension force	Enhanced osteogenic differentiation in a frequency-dependent manner	EYA1, SALL gene	–	^126^
PDLSC	Stretch (Flexcell-FX-6000-Tension System)	Osteogenic differentiation	Downregulation of SNHG8 and EZH2	Male Wistar rats	^130^
PDLSC	Cyclic mechanical stretch	Osteogenic differentiation	Downregulation of miR-146a and miR-34a, overexpression of CELF3	–	^24^
PDLSC	Mechanical force (Flexcell^®^ FX-5000™ Tension System)	Osteogenic differentiation	lncRNA-miRNA-mRNA	–	^132^
PDLSC	Dynamic tension	Osteogenic differentiation	LncRNA/circRNA-miRNA-mRNA networks	–	^133^
PDLSC (from orthodontic and periodontitis patients)	Static mechanical strain	m6A modification of RNA	–	–	^134^
PDLSC	Cyclic mechanical stretch	Enhanced osteoblast differentiation	Nrf2 activation	Male Wistar rats	^135^
PDLSC	Cyclic mechanical stress	Enhanced osteoblast differentiation	PI3K/AKT signaling pathway	Male Wistar rat	^136^
PDLC	Uniaxial cyclic tensile stress	Osteogenic differentiation	ROCK-TAZ pathway	–	^137^
PDLSC	Cyclic tensile stress	Osteogenic differentiation	Inhibited mmiR-129-5p expression, BMP2/Smad signaling pathway	–	^131^
PDLC	Cyclic stretch	Osteogenic differentiation	YAP/WNT5A/FZD4	–	^138^
PDLSC	Cyclic stretch	Promoted proliferation and osteogenic differentiation	Exosomes, miR-181b-5p/PTEN/AKT, BMP/Runx2	–	^139^
PDLF	Tension force	Osteogenic differentiation	PDGF-BB/PDGFRβ signals, JAK2/STAT3 pathway	Male Sprague-Dawley rats	^140^
PDLSC	Static tension	Osteogenesis	Autophagy	–	^141^
-	-	Osteogenesis	PIEZO1 channel activation	Male Sprague-Dawley rats	^142^

Mechanical force is transmitted to PDL cells and induced subsequent biological responses by various signaling pathways. Animal experiments have been done to verify these processes

*PDLF* periodontal ligament fibroblast, *PDLSC* periodontal ligament stem cell, *PDLC* periodontal ligament cell

### Compressive force

The biomechanical interaction between compressive force and PDLCs may involve a variety of signal proteins, ion channels, and other related signal transduction mechanisms. Initially, PDLCs exhibit changes in shape under compressive forces, accompanied by increased cell mortality, cell cycle arrest, and reduced proliferation.^107^ This response may be attributed to the inhibition of PDLC proliferation through the downregulation of the MIR31HG gene.^108^ Additionally, the Yes-associated protein (YAP) plays a crucial role in regulating PDLC behavior in response to compressive forces. Under intermittent compressive forces, YAP promotes the osteogenic differentiation of PDLCs.^109^ Conversely, under static compressive forces, YAP enhances inflammation and promotes osteoclast differentiation by upregulating the growth differentiation factor 15 (GDF15) gene.^110^ In addition to YAP, other proteins such as heat shock protein (HSP) can regulate bone remolding of PDL as well. For example, phosphorylation of HSP27 may inhibit aseptic inflammation in PDLCs induced by compression and suppress osteoclastic differentiation.^111^ Toll-like receptor 4 (TLR4) has also been identified as a potential receptor in the mechanotransduction of PDLCs. TLR4 regulates the phosphorylation of AKT and MAPK signaling pathways, which contributes to aseptic inflammation in PDLCs and affects bone remodeling.^112^ Additionally, ion channels like PIEZO1 may be involved in regulating osteoclast differentiation and apoptosis mediated by periodontal fibroblasts (PDLFs).^113,114^

Macrophages are key cells in bone resorption induced by mechanical force, associated with inflammation mediation and osteoclast differentiation.^115^ Static compressive force can promote the proliferation of periodontal ligament stem cells (PDLSCs) by activating TRPV4, causing osteogenic differentiation inhibition, macrophage migration, and osteoclast differentiation.^116^ Compressive force can also promote the production of H_2_S by PDLSCs and the polarization of M1 macrophages through STAT1 signaling pathway.^117^ The autophagy of PDLSCs and further inhibition of AKT signaling pathway resulting from compressive force are another possible mechanism of M1 macrophage polarization.^118^ Integrin-linked kinases (ILK) regulate PDLC autophagy in a PI3K-dependent manner,^119^ while lncRNA FER1L4 has been identified as a promoter of PDLSC autophagy via the AKT/FOXO3 signaling pathway.^120^ Moreover, exosomes secreted by PDLCs can promote the polarization of M1 macrophages through YAP-TEAD axis under compression, suggesting another mechanism of the compression-PDL-M1 macrophage polarization.^121^

Extracellular vesicles (EVs) including exosomes also participate in the mechanotransduction of PDLCs. Zhao et al.^121^ observed that compressive forces inhibit exosome secretion in PDLCs, likely due to increased apoptosis. Nonetheless, overall EV secretion by PDLCs is elevated under compressive forces.^122^ The inhibition of EV secretion is associated with reduced OTM, suggesting a new link between PDLC behavior and OTM.^122^ This relationship may involve enhanced expression of the PDL-derived exosome protein ANXA3, which promotes osteoclast differentiation through the ERK signaling pathway.^123^ In general, the role of PDLC-exosome interactions under compressive forces warrants further investigation.

**Fig. 3:**
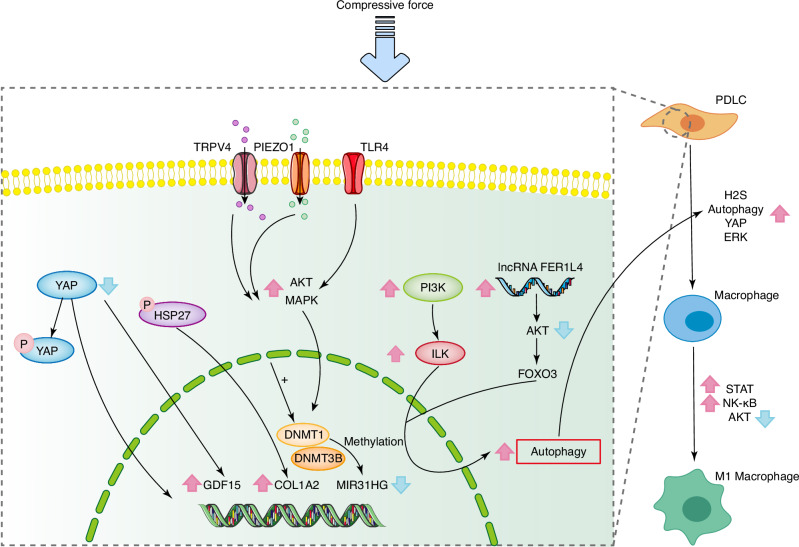
Molecule mechanisms of biomechanical signal transduction in PDLCs under compressive force

The biological responses to compressive force are complicated (Fig. 3). Compressive forces can regulate various cellular processes in PDLCs, including proliferation, apoptosis, autophagy, and EV secretion. These processes subsequently influence osteoclast and osteoblast differentiation, as well as macrophage behavior. However, excessive compressive forces can have detrimental effects, leading to root resorption and inflammatory bone destruction, potentially associated with increased expression of IL-6 in PDLCs and polarization of Th17 cells.^124^ Therefore, precise control of orthodontic forces is essential to ensure effective and safe OTM.

### Tensile force

Similarly, tensile force can also affect the behavior of PDLCs. Transcriptomic analyses have demonstrated that tensile forces alter the expression profiles of key genes in PDL interstitial cells, identifying six genes significantly associated with OTM.^125^ The effects of tensile force are modulated by its magnitude, frequency, and duration.^126,127^ Specifically, lower tensile forces tend to promote osteogenesis, inhibit inflammation, and facilitate OTM.^127^ Within the frequency range of 0.1–0.7 Hz, increased tensile frequency enhances the osteogenic differentiation of PDLSCs, potentially through genes such as EYA1 and SALL1.^126,128^

RNA plays a crucial regulatory role in PDLC behavior. Research indicates that tensile forces can alter the expression profiles of lncRNA and mRNA in PDLCs.^129^ For instance, tensile forces downregulate lncRNA SNHG8, which in turn decreases EZH2 expression and promotes osteogenic differentiation of PDLCs.^130^ Cyclic tensile forces also downregulate miR-36a and miR-146a, thereby enhancing PDLSC osteogenic differentiation by targeting CELF3.^24^ Additionally, cyclic stretching inhibits miR-129–5p expression, promoting osteogenesis through activation of the BMP2/Smad signaling pathway.^131^ Some scholars further proposed that the LncRNA/circRNA-miRNA-mRNA network participated in the regulation of osteogenic differentiation of PDLSCs under tension.^132^ Since lncRNA and circRNA act as sponges for miRNA and compete with mRNA, this network is known as the competitive endogenous RNA (ceRNA) network.^133^ The methylation of m6A plays a regulatory role in this process and is different in PDLSCs under periodontitis and healthy environment.^134^

In addition to RNA-mediated mechanisms, other factors contribute to osteogenic differentiation under tensile forces. Nuclear factor erythroid-2-related factor-2 (Nrf2) has been shown to facilitate osteogenic differentiation of PDLSCs under cyclic mechanical stretching, potentially through the PI3K/AKT signaling pathway.^135,136^ Transcriptional co-activator with PDZ-binding motif (TAZ) accumulates in the nucleus and interacts with core-binding factor α1 (Cbfα1, also known as RUNX2) to promote osteogenic differentiation of PDLCs, a process regulated by ROCK signaling.^137^ The YAP/WNT5A/FZD4 axis also plays a role in regulating PDLC osteoblast differentiation under cyclic stretching.^138^ Moreover, miR-181b-5p/PTEN/AKT signaling pathway participates in the osteocyte-exosome-mediated osteogenic differentiation of PDLCs.^139^ PDGF-BB/PDGFRβ signals promotes osteogenic differentiation through activation of JAK2/STAT3 pathway.^140^ Autophagy also significantly contributes to osteogenic differentiation under tensile forces.^141^ Additionally, the ion channel PIEZO1, which is activated under tensile forces, supports osteogenic differentiation on the tensile side.^142^

**Fig. 4:**
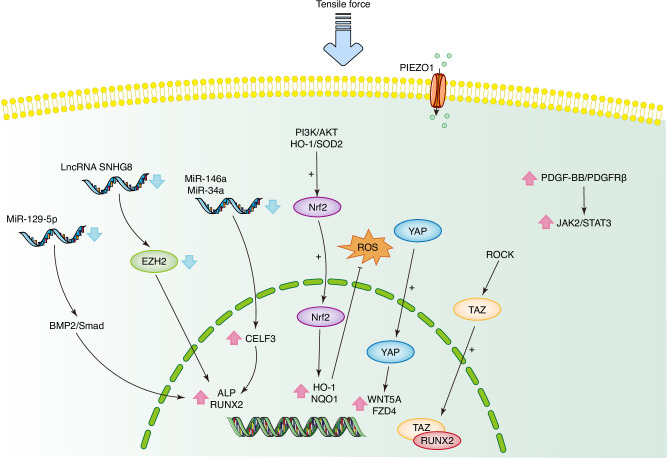
Molecule mechanisms of biomechanical signal transduction in PDLCs under tensile force

As summarized above, various molecules and signaling pathways are implicated in the biological responses of PDLCs to tensile forces (Fig. 4). Typically, tensile forces enhance osteogenic capacity in PDLCs, potentially laying the groundwork for bone formation on the tensile side.

## Structure-mechanics-mechanotransduction interplay in PDL

Structure, mechanics, and mechanotransduciton are inseparable components of OTM biomechanics. PDL’s structure serves as the foundation for its mechanical properties. As previously mentioned, mechanical properties vary across different regions of the PDL, reflecting their distinct internal structures. Mechanical forces may influence the PDL’s structure, such as through ECM remodeling. Additionally, structural factors influence cellular behaviors via mechanotransduction.

In vitro experiment demonstrated that the extracellular environment, in which cells reside, affects their biological properties via various structural factors. Cells cultured in a 2-D model exhibit different phenotypes compared to those in a 3-D model.^143^ The same cell type in a 3-D model shows a stronger response to mechanical stimuli over time than in a 2-D model, providing a better simulation of the in vivo environment.^144^ Moreover, cellular functions depend on the specific characteristics of the 3-D model used. For instance, nanofiber or porous structures appear to be more suitable for cells subjected to mechanical stress than microfiber structures.^145^ In addition to cultivation patterns, structural properties such as alignment and curvature also impact cell behaviors. Nano-groove have been reported to induce intracellular forces and promote osteogenesis, while micro-groove structures do not exhibit the same effects. The reason for this is that nano-groove facilitate adhesion induction, leading to the formation of cell attachments, whereas micro-grooves merely constrain space and limit cell extension.^146^ Curved structures that mimic the ECM can generate cytoskeleton tension and promote cell bridge formation, subsequently leading to osteogenesis through the influence of intracellular forces.^147^ Throughout these processes, intracellular forces connect environmental physical changes to nuclear biomechanics.^148^ Therefore, biology, mechanics, and biomechanical signal transduction are interdependent and influence each other, forming a network that mediates OTM.

## Conclusion

Periodontal ligament (PDL) is a fibrous connective tissue located between the root and alveolar bone, serving the critical function of transmitting and dispersing occlusal forces.^149^ Especially in orthodontic tooth movement, PDL senses mechanical stimulation, transmits forces, initiates signal transduction in periodontal ligament cells, and orchestrates alveolar bone remodeling. Therefore, a thorough understanding of the PDL’s biological composition, structural characteristics, mechanical properties, and biomechanical signaling processes is essential.

The PDL is composed of various elements, including cells, fibers, blood vessels, and nerves, which collectively underpin its functional capacity. Historically, the macroscopic structure of the PDL has been well-documented. Recent research has increasingly focused on its microstructure. Microscopically, the PDL exhibits considerable heterogeneity, influenced by factors such as root morphology, mechanical stimulation, and other variables. This structural and compositional variability establishes the foundation for the PDL’s mechanical properties. Studies have demonstrated that these properties are affected by tooth position, specific regions within a single tooth, loading conditions, and environmental temperature. To better elucidate the mechanical behavior of the PDL, researchers have employed mathematical modeling and finite element analysis to simulate its mechanical responses and develop various constitutive models. Mechanical stimulation induces biological responses in PDL cells, known as biomechanical signal transduction or mechanotransduction. Compressive forces predominantly regulate osteoclast differentiation and also impact cell proliferation, migration, autophagy, and macrophage polarization. Conversely, tensile forces are crucial for promoting osteogenic differentiation. Despite extensive research into the underlying mechanisms, the diverse molecules and signaling pathways involved remain complex and not fully understood. Furthermore, the effect of the extracellular matrix microstructure on cell fate within the PDL under mechanical stimulation warrants further investigation.
